# Phytochemical Analysis, Cytotoxicity, and Antitrypanosomal, Antioxidant, and Anti-Inflammatory Activities of *Clausena anisata* Fruit, *Azadirachta indica* Leaf, and Stem Bark Extracts

**DOI:** 10.1155/japr/7509588

**Published:** 2024-11-27

**Authors:** Emmanuel Kofi Kumatia, Felix Kwame Zoiku, Prince Kyei Baffour, Anne Boakyewaa Anokye-Kumatia, Alex Asase

**Affiliations:** ^1^Department of Phytochemistry, Centre for Plant Medicine Research, Mampong-Akuapem, Ghana; ^2^Department of Epidemiology, Noguchi Memorial Institute for Medical Research, University of Ghana, Accra, Ghana; ^3^Department of Pharmacy, Komfo Anokye Teaching Hospital, Kumasi, Ghana; ^4^Plant Development Department, Centre for Plant Medicine Research, Mampong-Akuapem, Ghana

**Keywords:** Alamar blue, cytotoxicity, DPPH, HepG2, neem, protein denaturation assay

## Abstract

Human African trypanosomiasis (HAT) is an infectious disease which kills humans and animals as a result of hematological distortions, oxidative stress, tissue and neuroinflammations. This study reports on the differences in cytotoxicity, antitrypanosomal, antioxidant, and anti-inflammatory activities of ethanol extracts from *Clausena anisata* fruit (CFE), *Azadirachta indica* (neem) leaf (NLE), and stem bark (NSBE), medicinal plants used to treat HAT in its endemic communities. The extracts remarkably inhibited *Trypanosoma brucei brucei* (GUTat 3.1) parasite in vitro with CFE recording the highest effect with an IC_50_ of 0.0055 (0.0955) *μ*g/mL. The IC_50_ of the standard *Coptis japonicum* was 0.5957 (0.0693) *μ*g/mL. Also, the antitrypanosomal activity of NLE was 123.34% higher than that of NSBE. The percentage number of wells containing viable *T. b. brucei* parasites was very significantly (*p* < 0.001) reduced for all the extracts after 48 h of incubation. Furthermore, the extracts did not show cytotoxicity against the liver (HepG2) cells (CC_50_s > 100 *μ*g/mL and SI = 13.12–32,025.45). NSBE contained the highest quantity of phenolic compounds and flavonoids and also produced the highest antioxidant and anti-inflammatory activity in the DPPH free radical scavenging assay (IC_50_ = 4.99 ± 0.018) and protein denaturation assay (IC_50_ = 0.1805 ± 0.0002 *μ*g/mL). In addition, phytochemical analysis showed that NLE contained the highest number of classes of phytochemical constituents (seven) among the extracts. These results indicate that CFE, NLE, and NSBE possessed significant antitrypanosomal activity as a result of their antioxidant and anti-inflammatory actions. However, a different mechanism was also involved in the antitrypanosomal activity of CFE and NLE, since their antitrypanosomal activity is greater than NSBE which demonstrated the highest antioxidant and anti-inflammatory activities. Due to the remarkable antitrypanosomal action of CFE, its constituents are being isolated for possible development into novel antitrypanosomal agents.

## 1. Introduction

Human African trypanosomiasis (HAT) which is commonly called sleeping sickness is a protozoan disease transmitted to humans and animals through the bite of tsetse fly [1]. The disease is contracted through infection with the protozoan species *Trypanosoma brucei* with which there are three subspecies, namely, *Trypanosoma brucei gambiense*, *Trypanosoma brucei rhodesiense*, and *Trypanosoma brucei brucei*. *T. b. gambiense* and *T. b. rhodesiense* infect humans only whereas *T. b. brucei* also infects and causes disease in animals and not humans [2, 3].

*T. b. gambiense* is the most widespread and contagions form of HAT accounting for 92% of all reported cases with *T. b. rhodesiense* being responsible for the extra 8%. HAT is endemic to rural areas of sub-Saharan Africa, and it leads to death if not treated with the appropriate medications [1].

Oxidative stress and harmful substances produced by the trypanosome parasite during infection lead to anemia, low platelet and WBC levels, tissue injury and inflammation, enlarged spleen, and loss of weight [4]. Furthermore, in some cases of both human and animal trypanosomiases, the parasite crosses the CNS and enters the brain which results in neuroinflammation [3, 5].

However, the drugs presently available for the treatment of the disease are toxic [6], few, and expensive. Furthermore, the diagnosis and treatment of HAT are complex and need specialized skills [1]. Therefore, most people in endemic environments of HAT rely on medicinal plants as their sources of treatment. Plants with polyphenols including flavonoids act as antioxidants and anti-inflammatory agents which counter oxidative stress and ameliorate tissue damage and inflammation [7]. Polyphenol consumption also prevents the body from developing several illnesses like neurodegenerative disorders, cancers, cardiovascular diseases, diabetes, inflammatory diseases, and infectious diseases [8]. *Clausena anisata* and *Azadirachta indica* are some of the medicinal plants employed in the treatment of HAT in sub-Sahara Africa.

*C. anisata* (Wild) Hook f. ex. Benth (Rutaceae) is a small to medium-size tree which grows up to the height of 10 m on the borders of forests in Africa. The leaves of the tree are 10–17 opposite or alternate leaflets which are pinnate compound with a terminal one which produced strong aniseed-like odor when crushed [9]. The tree produces green drupe-like fruits which turn dark wine to purple when ripped. The leaf, stem, and root of *C. anisata* are employed in traditional medicines as treatment for many human ailments such as infectious diseases (e.g., trypanosomiasis, schistosomiasis, leprosy, syphilis, gonorrhea, and malaria), noncommunicable diseases (e.g., diabetes, hypertension, and stroke), pain, inflammation, arthritis, and madness [10–14]. Pharmacological studies conducted on the plant showed that the leaf, stem, or root and their isolated constituents possessed analgesic, anti-inflammatory, antimalarial, antihypoglycemic, anticonvulsant, and antitrypanosomal activities [15–19]. However, no study has been conducted on the fruits of *C. anisata.*

*A. indica* (A. Juss), Meliaceae, which is commonly called neem, is an evergreen tree with a height of 15–30 m on maturity. *A. indica* tree has a bulky crown which extends from 10 to 20 m wide [9, 20]. The branches have opposite and simply pinnate leaves, which are 20–40 cm in length, crowding near the apex of the branches. The tree bears small green to greenish-yellow seeded drupe, ellipsoidal fruits which turn yellow upon ripping [20]. The *A. indica* tree grows mostly in Africa and Asia where its different parts have been used in folkloric medicine to treat diseases such as trypanosomiasis, malaria, hypertension, cardiovascular diseases, cancers, and diabetes [9, 21]. Several scientific investigations were conducted on the various parts of the *A. indica* tree which revealed that it possessed antimalarial, antimicrobial, antioxidant, anti-inflammatory, antidiabetic, and anticancer activities [22–25].

The leaf and the stem bark of the *A. indica* tree were also shown to demonstrate antitrypanosomal actions [16, 26, 27]. However, the antitrypanosomal activity of the tree's leaf and stem bark had not been compared with each others to determine which one is the best treatment for the disease. Furthermore, no medicinal property of *C. anisata* fruit has also been reported, although numerous biological activities were reported for the other parts.

Additioally, plants can be poisonous by their nature as a result of the chemical constituents they produce and/or the chemicals they absorb in their environments such as the soil, water, and air. This may lead to other health risk and/or death when they are consumed. The World Health Organization stated that “Safety is a fundamental principle in the provision of herbal medicines and herbal products for health care, and a critical component of quality control” [28]. It is therefore imperative to evaluate the toxicity of medicinal plants used in traditional medicines and their herbal medicinal products in other to ascertain their safety.

The aim of this study therefore is to evaluate and compare the antitrypanosomal, antioxidant, and anti-inflammatory activities of *C. anisata* fruit and *A. indica* leaf and stem bark extracts and to evaluate the safety of the extracts through cytotoxicity testing against liver (HepG2) carcinoma cell line.

## 2. Materials and Methods

### 2.1. Collection and Extraction of Plant Material

*C. anisata* and *A. indica* were identified and authenticated by the first author of this paper, who is a pharmacognosist at the Centre for Plant Medicine Research (CPMR), Mampong-Akuapim, Ghana. The ripe fruit of *C. anisata* (CF) was harvested in April, 2023, from the premises of the CPMR, Mampong-Akuapim, Ghana. Furthermore, the *A. indica* leaf and stem bark were also collected in March, 2023, alongside the Adukrom–Somaya road in the Eastern Region of Ghana. CF was rinsed with portable water and dried in the sun for 10 days. It was later dried in an oven at 60°C for 48 h. Approximately, 400 g of CF was pulverized into course powder and soxhleted with absolute ethanol (1 L × 3) for about 6 h each. Using the rotary evaporator, the extract was evaporated to dryness. A brown solid was obtained and labeled *C. anisata* fruit extract (CFE) (34.828 g; yield = 8.71%*w*/*w*).

### 2.2. Extraction of *A. indica* Leaf and Stem Bark

Cold maceration of 200 g of *A. indica* leaf was carried out for 4 days with 2 L of absolute ethanol each twice. The extracts were filtered, combined, and dried using a rotary evaporator at 48°C to obtain a dark green solid labeled *A. indica* leaf extract (NLE) (31.93 g; yield = 15.96%*w*/*w*). A similar procedure was applied to 200 g of the stem bark to produce dark reddish solid, *A. indica* stem bark extract (NSBE) (27.56 g; yield = 13.78%*w*/*w*).

### 2.3. Qualitative Phytochemical Screening of the Extracts

The classes of phytochemicals present or absent in the extracts were analyzed as described previously [29].

### 2.4. Quantification of Polyphenols

Total polyphenol constituents in the extracts were determined using the Folin–Ciocalteu method [30] with some modifications according to Kumatia, Baffour, and Bolah [31]. Briefly, 2 mL of 10% Folin–Ciocalteu reagent was added to 0.4 mL of 1, 0.5, 0.25, 0.125, 0.0625, 0.03125, 0.015625, and 0.0078125 mg/mL each of the extract and incubated in the dark for 8 min. The mixture was then mixed with 1.6 mL of 7.5% sodium carbonate solution in test tubes. This mixture was also incubated up to 90 min in a dark cabinet. The absorbance of the mixture was then recorded at 765 nm with a spectrophotometer (double-beam UV/VIS BK-D590, Shandong, China). Gallic acid, a standard polyphenol was run under parallel experimental settings to produce a calibration curve from which the quantity of polyphenols in the extracts was calculated as gallic acid equivalent (GAE) per milligram of dry sample. Each individual sample concentration was run in triplicate.

### 2.5. Evaluation of Antioxidant Activity

The DPPH free radical scavenging assay was employed to determine the antioxidant capacity of the extracts [19, 32]. About 4 mL of 0.2 mM, DPPH-methanol solution was combined with 2 mL each of 1, 0.5, 0.25, 0.125, 0.0625, 0.03125, 0.015625, or 0.0078125 mg/mL of the extract and incubated in a dark cabinet for 30 min. The absorbance was determined at 517 nm using a spectrophotometer (double-beam UV/VIS BK-D590, Shandong, China). Ascorbic acid solution and 2 mL of methanol in 0.2 mM of 2 mL DPPH solution were used under the same conditions as standard and blank, respectively. Each test was done in triplicate. The percentage inhibition was calculated as indicated below:

 $$\begin{array}{c} \%\text{Inhibition}=\frac{\text{absorbance of the blank}-\text{absorbance of test sample}}{\text{absorbance of the blank}}\times 100\% \end{array}$$

The log-normalized response curves were then plotted with the percentage inhibition values of the samples using GraphPad Prism 9.5.1. The antioxidant activities of the samples were calculated from the curves as the 50% minimum inhibitory concentration (IC_50_) values.

### 2.6. In Vitro Anti-Inflammatory Acclivity

Anti-inflammatory acclivity of the extracts was assessed using the in vitro protein denaturation assay according to Chandra et al. [33], with some amendments [19]. Briefly, 2 mL each of the extract at 3.90–2000 *μ*g/mL was mixed with 0.2 mL of flesh egg albumin and 2.8 mL of PBS (at pH 6.4). Incubation of the mixture was carried out for 10-min room temperature prior to it being heated for 20 min at 70°C. It was then allowed to cool, and its turbidity measured at an absorbance of 660 nm using the spectrophotometer. Diclofenac sodium salt was employed as the standard anti-inflammatory drug whereas the negative control was 10% DMSO/water (*v*/*v*). The percentage inhibition of denaturation protein was calculated as

 $$\begin{array}{c} \%\text{Inhibition}=\frac{\text{aborbance of negative control}-\text{absorbance of sample}}{\text{absorbance of negative control}}\times 100\% \end{array}$$

The log-normalized response curves were then plotted with the percentage inhibition values of the samples using GraphPad Prism 9.5.1. The 50% inhibitory concentrations (IC_50_) were obtained.

### 2.7. Preparation of the Test Solutions of the Plant Extracts

The extract (10 mg) was reconstituted in 1.0 mL of 50% ethanol to make a concentration of 10 mg/mL. The mixture was vortexed into a homogeneous solution in a 1.5-mL Eppendorf tube to obtain a stock solution. The stock solution was then transferred into a cultured blood and diluted to a concentration of 400 *μ*g/mL with medium after which the mixture was filtered into fresh sterile tubes.

### 2.8. Evaluation of Antitrypanosomal Activity

The culturing of the *T. brucei* (GUTat 3.1) cells and antitrypanosomal activity assays were performed according to methods described by Roz et al. [34] with some modifications. Details of the methods are described below.

#### 2.8.1. Preparation of *T. brucei* (GUTat 3.1) Cells

Approximately, 10 *μ*L of cells each was mounted in the four circular chambers (*A*, *B*, *C*, and *D*) on the Neubauer chamber to determine their concentration. The concentration of the cell per milliliter was then calculated using the following formula:

 $$\begin{array}{c} \frac{\left(A+B+C+D\right)}{4\times 10\text{ E}4\;} \end{array}$$

Cell concentration of 3 × 10^5^ cells/mL is then prepared from the stock solution using the dilution formula.

#### 2.8.2. Antitrypanosomal Activity Assay

Approximately, 50 *μ*L each of the extract at concentrations of 400, 200, 100, 50, 25, 12.5, 6.25, 3.125, and 1.5625 *μ*g/mL was prepared by twofold dilution from the stock extract solution and transferred into their various wells. A volume of 50 *μ*L of the trypanosomal cells (3 × 10^5^cells/mL) was then added to each well and incubated in darkness for 24 h at 37°C in 5% CO_2_. Thereafter, 10% Alamar blue (10 *μ*L in 100-*μ*L well content) was added to each well and incubated for another 24 h at 37°C in 5% CO_2_ in darkness. The absorbances of the plates were then recorded at 540 nm with a spectrophotometer. *Coptis japonicum* (*CJ*) extract was run alongside the extracts as positive control. The negative control contained only the media without any cells.

#### 2.8.3. Determination of IC_50_ Values

The results were analyzed using GraphPad Prism 8.0.1 software. The IC_50_ values were obtained from nonlinear regression curve of the dose-response equation using log (inhibitor) versus response (variable slop, four parameters).

#### 2.8.4. The Percentage Number of Wells Containing Live Trypanosome Parasites

The percentage number of wells containing live trypanosome parasites after 48 h of culture was calculated by counting the number of pink spots (wells containing live parasites) divided by total number of wells per extract (pink spots + blue spots (wells containing dead parasites)) and finding their percentage as shown:

 $$\begin{array}{c} \%\text{number of wells with live parasite}=\frac{\text{number of wells containing live parasites}}{\text{total number of wells containing live parasites}+\text{dead parasites}}\times 100 \end{array}$$

### 2.9. Determination of Cytotoxicity of the Extracts

#### 2.9.1. Cell Culture

This test was conducted by adapting the method of Ham et al. [35]. Liver (HepG2) cell line (RIKEN BioResource Centre Cell Bank, Japan) was cultivated in Dulbecco's modified Eagle medium (DMEM) complemented with 10% fetal bovine serum (FBS) (Sigma–Aldrich, Urbana, IL, United States) and 1% penicillin–streptomycin–L-glutamine (Sigma–Aldrich, Urbana, IL, United States). The cell was cultivated in a flask and preserved in a humidified incubator (Panasonic Healthcare Company Limited, Japan) at 37°C, provided with 5% CO_2_, then passaged on attaining approximately 90% confluency.

#### 2.9.2. Cytotoxicity Assay

The cytotoxic action of the extracts against the liver (HepG2) carninoma cell line was accessed by employing the 3-(4, 5-dimethylthiazol-2-yl)-2, 5-diphenyltetrazolium bromide (MTT) assay procedure as described by Appiah-Opong et al. [36]. The cells were seeded at a density of 1 × 10^5^ cells per well in 96-well plates. They were then augmented with 10% FBS and 1% penicillin–streptomycin–L-glutamine. Various concentrations of the reconstituted individual extracts at 100–6.25 ?g/mL or media alone (as negative control) were treated with the cells prior to their incubation at 37°C, 5% CO_2_ for 72 h. Around 20 mL of MTT solution (7.5 mg/mL) was mixed with each well after the incubation before reincubation of the plates at room temperature for 24 h. Formazan crystal formation in the wells was dissolved by addition of 200 mL acidified isopropanol. The absorbance of the mixture in each well was measured at 570 nm in a microplate reader (Tecan Infinite M200 PRO, Switzerland). All assays were conducted in triplicate. Percentage survival of cells at each concentration of an extract was determined using the following formula:

 $$\begin{array}{c} \text{Cell viability }\left(\%\right)=\frac{A_{0}-A_{1}}{A_{0}}\times 100 \end{array}$$

where *A*_0_ is the mean absorbance of wells only media (control) and *A*_1_ is the mean absorbance of wells treated with extracts. The concentrations at which 50% cytotoxic effect occurred (50% cytotoxic concentration (CC_50_) values) were then calculated by plotting the concentration of extract against the percentage cell viability to derive a dose–response curves.

### 2.10. Selectivity Indices (SIs)

The SI of the extracts was calculated according to Kumatia et al. [19] as

 $$\begin{array}{c} \text{SI}=\frac{\text{CC}50}{\text{IC}50} \end{array}$$

### 2.11. Statistical Analysis

The assays were conducted in triplicate for each extract concentration, and the IC_50_ results presented as $\bar{x}$ (SD), that is, mean (standard deviations) for the antitrypanosomal activity [19]. The quantitative phytochemical results were expressed as $\overset{®}{x}\pm \text{SEM}$.

## 3. Results

### 3.1. Qualitative Phytochemical Screening

The results of the qualitative phytochemical screening of the extracts are shown in Table 1. The NLE has more phytochemical constituents (seven) than its stem bark (six). The NLE and *A. indica* stem bark extract (NSBE) both have similar constitutes except alkaloids which are absent in NLE but present in NLE. Phytosterols were also present in NSBE but were not found in NLE, which has alkaloids. CFE also has five classes of phytoconstituents similar to those of NLE and NSBE except for the absence of triterpenes.

### 3.2. Qualitative Phytochemical Screening

The result of the phytochemical screening testes is shown in Table 1 below. NLE has the highest number (seven) of classes of chemical constituents. NSBE and CFE both have five classes each of chemical constituents.

NLE and NSBE differs in three classes of chemical constituents. While NLE has alkaloids and anthracenosides which were not present in NSBE, phytosterols were also seen in NSBE which were absent in NLE. Out of the five classes of chemical constituents in CFE, there are four classes of similar chemical constituents in NLE and NSBE, respectively, except alkaloids, triterpenes, or phytosterols.

### 3.3. Quantitative Phytochemical Analysis

The results of the quantitative phytochemical screening of the extracts are shown in Table 2. NSBE contained the largest quantity of polyphenols and flavonoids among the three extracts. On the other hand, NLE and CFE contained similar quantity of polyphenols and flavonoids (Table 2).

### 3.4. DPPH Free Radical Scavenging and Anti-Inflammatory Activities

The results of the antioxidant activity using the DPPH free radical scavenging and anti-inflammatory activities using the protein denaturation assay are shown below in Table 3.

NSBE produced the least IC_50_ in both the DPPH free radical scavenging assay (4.99 ± 0.018 *μ*g/mL) and the protein denaturation assay (0.1805 ± 0.0002 mg/mL), respectively. These values are almost the same as the IC_50_ produced by the standard antioxidant drug, Ascorbic acid (3.099 ± 0.123 *μ*g/mL), and anti-inflammatory drug, diclofenac sodium (0.1958 ± 0.00013 mg/mL), respectively (Table 3). The antioxidant and anti-inflammatory activities of NSBE are six times and three times higher than those of NLE, respectively. NSBE also produced six times higher antioxidant activity and two times higher anti-inflammatory activity than CFE.

### 3.5. Antitrypanosomal Assay, Cytotoxicity, and SI

**Figure 1:**
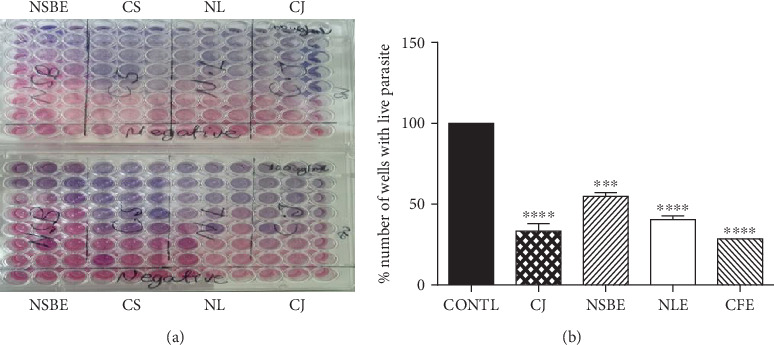
(a) Picture showing the effect of the extracts, standard, and negative control on the survival of *T. brucei brucei* (GUTat 3.1) parasite incubated with the extracts for 48 h with Alamar blue for 24 h out of the 48 h. Pink and blue colour spots indicate a well with live and dead parasites, respectively. (b) Percentage number of wells containing live parasites after incubation. CFE, *C. anisata* fruit extract; NLE, *A. indica* leaf extract; NSBE, stem bark extract; *CJ*, *C. japonicum*; CONTL, negative control. Note: In the picture above NSBE, CFE and NLE were referred to as NSB, CS, and NL, respectively. ⁣^∗∗∗^*p* < 0.001 and ⁣^∗∗∗∗^*p* < 0.0001 compared to viable parasites in control wells, one-way ANOVA followed by Dunnett's test.

The results of the antitrypanosomal activity of the extracts are shown in Figures 1(a) and 1(b) and Table 4 below. CFE gave the lowest number of wells (28.57 ± 0.00%) with surviving trypanosome parasites after 48 h of incubation. On the order hand, the control wells gave 100 ± 0.00% cell survival under the same conditions (Figures 1(a) and 1(b)).

The increasing order of percentage number of wells containing live parasites after incubation with extracts is CFE < *CJ* < NLE < NSBE < CONTL (Figure 1(b)). The increasing order of the IC_50_s produced by the extracts and standard agent also followed the same order described for percentage number of wells containing live parasites, with CFE which produced the lowest IC_50_ of 0.0055 *μ*g/mL (Table 4).

## 4. Discussions

Three important indicators are considered in in vitro efficacy evaluation of test substances such as crude extracts, compounds, and drugs, in order to determine the efficacy, toxicity, and therapeutic potential of the test substance. They are the IC_50_, CC_50_, and SI of the test substance.

The IC_50_ is the concentration of a substance required to produce half the effect that substance generates in a biological system. The lower the IC_50_, the lesser the amount of the extract or drug required to produce the desired effect, hence, the higher the activity of the extract or drug. In this study, CFE gives the lowest IC_50_ value. Therefore, CFE demonstrated the highest antitrypanosomal activity among the tested substances. Furthermore, NLE also shows 55.24% higher antitrypanosomal activity than NSBE (Table 4). Atindehou et al. [37] reported that the in vitro antitrypanosomal activity of medicinal plant extract is grouped as inactive, weak, or good when the IC50 value of the extract in antitrypanosomal assay is ≥ 25 *μ*g/mL, between 25.0 and 8.0 *μ*g/mL, or < 8.0 *μ*g/mL, respectively. Therefore, CFE, NLE, and the standard antitrypanosomal agent, *CJ* (having an IC_50_ of 0.0055–6.54 *μ*g/mL), possessed very good antitrypanosomal activity. On the other hand, the antitrypanosomal activity of NSBE in this experiment is weak.

Furthermore, resazurin, the fluorescent indicator dye with blue colour in Alamar blue reagent, is reduced to resorufin which is pink in colour, as a result of metabolic activities of live trypanosome parasites present in the culture [38]. The number of wells containing viable parasites after incubation with the extracts/drug therefore is directly proportional to the concentration of viable/live parasites and the intensity of the pink colour. This is to say that the percentage number of wells with viable trypanosome parasites after incubation with extract/drug is inversely proportional to the antitrypanosomal activity of the extract. The results show that the percentage number of wells with viable trypanosome parasites for the extracts was in an increasing order of CFE < *CJ* < NLE < NSBE < CONTL (Figure 1(b)). This confirmed that the increasing order of antitrypanosomal activity of the extracts is in the reverse order as: CONTL < NSBE < NLE < *CJ* < CFE, confirming the IC_50_ results (Table 4).

Several investigators had shown that various parts of the *A. indica* possessed antitrypanosomal activity. Mbaya et al. [39] showed that the ethanolic extract of *A. indica* stem bark had an LD_50_ of 870 mg/kg via intraperitoneal injection and produced significant in vitro and in vivo ant-trypanosomal activity against *T. b. brucei*. The extract prevented the development of parasitemia at 100 mg/kg vivo and reversed reduction in packed volume (PCV) after infection in rats. Tauheed et al. [40] also reported that partially purified chromatographic fractions of *A. indica* leaf significantly inhibited trypanosome alternative oxidase (TAO) and suppressed parasite motility with EC_50_ reaching to as low as 0.005 and 0.004 *μ*g/*μ*L. Other studies showed that *A. indica* stem bark and leaf possessed in vivo antitrypanosomal activity by reducing parasitemia and improving packed cell volumes [26, 27]. Kamte et al. [16] also reported that the essential oil from *A. indica* leaf possessed antitrypanosomal activity with IC_50_ of 15.21 ± 0.97 *μ*g/mL. However, the results from our study showed that the cold macerated extract of the leaf of *A. indica* (antitrypanosomal IC_50_ = 6.54 *μ*g/mL) has a better antitrypanosomal activity than the essential oil extracted from the *A. indica* leaf.

Although different studies reported the antitrypanosomal activity of *A. indica* leaf and stem bark, separately, none compared the antitrypanosomal activity of these two parts of *A. indica* in order to show which part is the best antitrypanosomal agent, to the best of our knowledge. This study has been able to show that the 70% ethanol extract of *A. indica* leaf, possessed better antitrypanosomal activity compared to the same extract from the stem bark of *A. indica*.

Increase levels of oxidants which lead to oxidative stress are one of the major characteristics of trypanosomal infection [41]. The trypanosome parasite produces sialidase and phospholipase which destroy the membranes of red blood cells (RBCs) resulting in the release of reactive oxygen species (ROS) that generate oxidative stress and lipid peroxidation within the RBC [42].

Antioxidant defense mechanism functions by inhibiting the initial production of free radicals, neutralizing and oxidizing them into less toxic compounds, inhibiting the secondary production of toxic metabolite or inflammatory mediators, stopping chain generation of secondary oxidants, and reforming the molecular injuries induced by the free radicals [43].

Medicinal plants with in vitro antioxidant activity are also known to produce corresponding in vivo antioxidant action [7], hence mopping up ROS and reversing their destructive effects. The results indicate that the extracts possessed good hydrogen or electron donating capacity to reduce the DPPH free radical to its stable state with relatively low IC_50_ values ranging from 4.99 ± 0.018 to 31.09 ± 0.893 *μ*g/mL which are closed to those of the standard antioxidant compound, ascorbic acid (3.099 ± 0.123 *μ*g/mL). Therefore, the extracts possessed remarkable antioxidant activity which contributes to their antitrypanosomal activity.

Trypanosomiasis infection leads to inflammation of some organs and tissues in the body as a result of activation of the body's immune system, which contributes to the severity of the disease and death. Neuroinflammation was also observed as one of the patterns in postmortem specimen taken from dead bodies of HAT patients with features such as diffuse encephalomeningitis and inflammatory infiltrate consisting of lymphocytes, macrophages, and plasmacytes [5, 44]. Neuroinflammation in trypanosome infection was also reported in rodent and primate models of experimental trypanosomiasis [45, 46]. For this reason, we assessed the ability of the extracts to overcome inflammation during trypanosomiasis using the protein denaturation assay. The results indicate that NLE, NSBE, and CFE possessed remarkable anti-inflammatory activity with very low IC_50_ values (0.1805 ± 0.0002–0.5457 ± 0.0009) which are similar to those of the standard anti-inflammatory drug, diclofenac sodium (0.1958 ± 0.0001 mg/mL). This shows that the extracts also exert their antitrypanosomal activity via modulation of the inflammation reaction.

Plants which contain flavonoids and other phenolic compounds are very potent antioxidant and anti-inflammatory agents [7]. Some studies have shown that plant extracts with higher quantity of phenolic compounds and flavonoids produce the most antioxidant and anti-inflammatory activities [47, 48]. That was the case in this study, such that NSBE which has 622.11%–971.34% phenolic compounds and 912.05%–985.82% flavonoid contents, respectively, than NLE and CFE also produced about 567.94%–623.05% antioxidant activity and 197.01%–302.32% anti-inflammatory activity than the NLE and CFE. Therefore, it was expected that NSBE should also be the extract with the highest antitrypanosomal activity. However, CFE and NLE rather demonstrated far higher antitrypanosomal activity than NSBE. This indicates that the mechanism of antitrypanosomal action of the extracts is not only limited to antioxidant and anti-inflammatory activities but is also due to a different mechanism which overrides the antioxidant and anti-inflammatory activities.

The CC_50_ of an extract or a drug is the concentration of the drug or the extract which can decrease the viability of cells by 50% compared to the control without the extract or drug. The CC_50_ is therefore, a measure of cellular toxicity of a test substance. According to Lima, Silva, and Melo [49], a test compound is classified as noncytotoxic, moderately cytotoxic, and cytotoxic if it produces a CC_50_ greater than 90 *μ*g/mL, 2–89 *μ*g/mL, or less than 2 *μ*g/mL, respectively. In this study, the CC_50_ values obtained for the ethanol extract of *C. anisata* fruit (CFE), *A. indica* leaf (ALE), and stem bark (NSBE) against the human liver HepG2 cells were greater than 100 *μ*g/mL. This indicates that the extracts are not toxic to the liver cells.

SI is the ratio of the CC_50_ to the IC_50_. The higher the SI of an extract or drug, the higher its efficacy and lesser its toxicity. In general, test agents with SI greater than or equal to 10 are considered to be very efficacious with less cytotoxicity (cell toxicity) [50]. These substances are therefore considered good and safe therapeutic agents. Our results show that the SI of the three extracts were greater than 10, especially CFE which produced a gargantuan SI of 32,025.45. Hence, the ethanol extracts of *C. anisata* fruit and *A. indica* leaf and stem bark are good and safe therapeutic agents against HAT.

## 5. Conclusion

This study has shown for the first time that the 70% ethanol Soxhlet extract of *C. anisata* fruit (CFE) possessed considerable antitrypanosomal activity. Furthermore, the cold ethanol extract of *A. indica* leaf possessed better antitrypanosomal activity than NSBE produced by the same method. The three extracts also displayed high degrees of anti-inflammatory and antioxidant activities in vitro and did not show cytotoxicity against HepG2 cell lines. These findings justified the use of these plants for the treatment of trypanosomiasis. It further shows that *C. anisata* fruit, *A. indica* leaf, and stem bark contained chemical constituents which should be isolated and studied for development into antitrypanosomal dugs. The chemical constituents in *C. anisata* fruit are being isolated for characterization and antitrypanosomal studies.

## Figures and Tables

**Table 1:** Phytochemicals present and absent in the extracts.

**Class of phytochemical**	**NLE**	**NSBE**	**CFE**
Reducing sugars	+	+	+
Phenolic compounds	+	+	+
Polyuronides	−	−	−
Saponins	+	+	−
Cyanogenic glycosides	−	−	−
Alkaloids	+	−	+
Flavonoids	+	+	+
Triterpenes	+	+	−
Phytosterols	−	+	+
Anthracenosides	+	−	−

*Note:* present (+); absent (−).

**Table 2:** Amount of phenolics compounds and flavonoids present in the extracts.

**Samples**	**Total phenolic content (mg/100 mg) of GAE**	**Total flavonoid content (mg/100 mg) of QE**
NLE	9.544 ± 0.525	6.276 ± 0.401
NSBE	59.35 ± 0.331	61.91 ± 0.994
CFE	6.108 ± 0.169	6.788 ± 0.518

*Note:* Values are mean ± SEM, *n* = 3.

Abbreviations: AcA, ascorbic acid; CFE, *Clausena* fruit extract; DfS, diclofenac sodium; NLE, *A. indica* leaf extract; NSBE, *A. indica* stem bark extract.

**Table 3:** In vitro antioxidant and anti-inflammatory activities of the extracts.

**Samples**	**DPPH assay: ** **I** **C**_50_ ± **SEM (*μ*g/mL)**	**Protein denaturation assay: ** **I** **C**_50_ ± **SEM (mg/mL)**
AcA	3.099 ± 0.123	—
DcS	—	0.1958 ± 0.00013
NLE	28.34 ± 1.021	0.5457 ± 0.0009
NSBE	4.99 ± 0.018	0.1805 ± 0.0002
CFE	31.09 ± 0.893	0.3556 ± 0.0016

*Note:* Values are mean ± SEM, *n* = 3.

Abbreviations: AcA, ascorbic acid; CFE, *Clausena* fruit extract; DfS, diclofenac sodium; NLE, *A. indica* leaf extract; NSBE, *A. indica* stem bark extract.

**Table 4:** Results of the IC_50_ of the antitrypanosomal assay, CC_50_ of the cytotoxicity, and SI.

**Extract**	**IC**_**50**_ ** (*μ*g/mL)**	**CC**_**50**_ ** (*μ*g/mL)**	**SI**
CFE	0.0055 (0.0955)	176.14	32,025.45
NLE	6.54 (0/066)	100.19	15.32
NSBE	14.61 (0.0677)	191.71	13.12
*CJ* (standard)	0.5957 (0.0693)	Nd	Nd

*Note:* The IC_50_ is expressed as mean (SD), *n* = 3.

Abbreviations: CFE, *C. anisata* fruit extract; *CJ*, *C. japonicum*; Nd, not determined; NLE, *A. indica* leaf; NSBE, stem bark extract.

## Data Availability

Data is available on request from the authors.
